# Concept-based reasoning in medical imaging

**DOI:** 10.1007/s11548-023-02920-3

**Published:** 2023-05-25

**Authors:** Anuja Vats, Marius Pedersen, Ahmed Mohammed

**Affiliations:** 1 Department of Computer Science, NTNU, 2815 Gjøvik, Norway; 2 SINTEF Digital, 0373 Oslo, Norway

**Keywords:** Interpretability, Biomedical imaging, Capsule endoscopy, Deep learning

## Abstract

****Purpose**:**

As concept-based reasoning for improving model interpretability becomes promising, the question of how to define good concepts becomes more pertinent. In domains like medical, it is not always feasible to access instances clearly representing good concepts. In this work, we propose an approach to use organically mined concepts from unlabeled data to explain classifier predictions.

****Methods**:**

A Concept Mapping Module (CMM) is central to this approach. Given a capsule endoscopy image predicted as abnormal, the CMM’s main task is to identify which concept explains the abnormality. It consists of two parts, namely a convolutional encoder and a similarity block. The encoder maps the incoming image into the latent vector, while the similarity block retrieves the closest aligning concept as explanation.

****Results**:**

Abnormal images can be explained in terms of five pathology-related concepts retrieved from the latent space given by inflammation (mild and severe), vascularity, ulcer and polyp. Other non-pathological concepts found include anatomy, debris, intestinal fluid and capsule modality.

****Conclusions**:**

This method outlines an approach through which concept-based explanations can be generated. Exploiting the latent space of styleGAN to look for variations and using task-relevant variations for defining concepts is a powerful way through which an initial concept dictionary can be created which can subsequently be iteratively refined with much less time and resource.

Introduction

When machine learning models are utilized for critical applications such as severity grading in ulcerative colitis in a patient, the clinician may want to understand what aspects within the endoscopic image led the machine to decide so. Although through the use of feature attribution methods [1] it may be possible to highlight which parts of the image show abnormality, clinicians may prefer to reason among each other in terms such as: increased mucosal “inflammation” causes a given severity. Not only clinicians, but humans in general understand through “concepts” that are high-level ideas easily interpretable and translatable within the domain. For example, the concept “fins”: the presence implies a fish and vice versa. It has been found that patients prefer easily understandable explanations catering to their specific case and it improves a patient’s trust in the model. Similarly, doctors may find visual information such as the explanations produced by LIME [2] difficult to interpret. They usually prefer clinical context around explanations [3] such as explanations that provide biomedical links for the decisions. In this regard, concept-based explanations are at the level that is natural to and easily understandable by the end users. Further, explanations are more relatable and actionable when presented in terms of concepts of the domain. Recent work promotes techniques to enable interpretation of decisions beyond low-level features (as pixels) so that outputs can be understood in terms of human-understandable concepts [4]. This requires the concepts, in terms of which the outputs are to be attributed, to be sufficiently well defined. For example, labeling all images with blue eyes as belonging to the “blue-eye” concept and so on. Once defined, concepts can be learnt through examples during training and attributed to during inference [4]. Usually, the activation of one of the layers of a binary classifier trained on concept and non-concept examples is used to calculate a concept activation vector (CAV) and it can indicate whether an incoming sample belongs to a concept or not [4]. As many binary classifiers as the number of concepts are required in this case as each CAV specializes in one concept. If such exemplar samples are not directly obtainable through visual inspection, prior information from experts must be used to collect such samples and calculate CAVs [5]. However, such manual curation of concepts is not so trivial [6]. Consider the label scarce domain of wireless capsule endoscopy with availability of large but un-annotated dataset. Here, the curation of concepts would not only be time-consuming, but can be done only by a medical expert. Similarly, consider the concept of faulty versus robust heavy machinery design which can only be reliably identified by a machine engineer. This is a bottleneck to concept-based learning for many domains including medical, where domain knowledge is essential to concept definition. This leads to the question investigated in this work: for domains where concept definition is an arduous exercise, how could we mine the concepts underlying the data without explicit supervision and generate useful explanations? Further, the unique challenges of capsule endoscopy make it an interesting domain to inquire this. Finding pathologies in capsule endoscopy can be challenging due to distortions such as illumination variation, rotational and motion blur as well as occlusions due to floating gastrointestinal content. Considering that some pathologies such as lesions and ulcers can be small scale, identifying pathologies against a dynamic canvas of gastrointestinal variations can be challenging.

The aim of disentanglement learning is that the factors underlying the data can be captured independently [7]. In this regard, the latent space of generative models has been shown to exhibit a degree of disentanglement between latent dimensions. These dimensions correspond to visual concepts such as absence or presence of inflammation in endoscopic image and can be selectively integrated in the learning framework for enhancing interpretability. In fact, mapping of latent dimensions to human concepts is not new and has been discussed in the context of image editing [8] as well as language understanding [9]. In this work, we show how certain latent dimensions of style-based generative adversarial networks (StyleGANs) [10] directly translate as task-relevant concepts and propose a simple framework for generating concept-based explanations for a classifier.

## Methodology

We train StyleGAN2 [10] on wireless capsule endoscopy images and perform semantic factorization [11] in the latent space $\mathcal {W} \in \mathcal {R}^{512}$ to obtain 512 candidate concepts. Not all candidate concepts are task-relevant, for example, in abnormality classification, concepts pertaining to pathology (inflammation, ulcer, polyp) are required as opposed to those of anatomy (small or large intestine) or image clarity (clear or blurred due to capsule motion). However, once the data are decomposed into candidate concepts, the pertinent concepts can be easily identified with involvement from medical experts by simply asking them to look at the images generated for each candidate concept and indicate those consistent with the task. The task-relevant concepts thus form a concept dictionary $\mathcal {D}$. Further, a classifier $\mathcal {C}$ outputs label $y\in \{0,1\}$ for input $x_i \in \mathbb {R}^n$ indicating whether the image is normal or abnormal. A concept mapping module (CMM) then explains $\mathcal {C}$ by identifying the underlying concept for $x_i$ from $\mathcal {D}$.

**Fig. 1:**
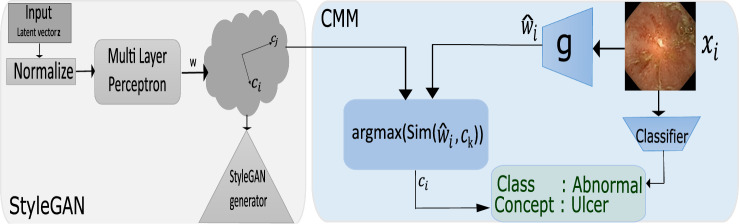
Using the latent space of a pretrained StyleGAN (gray), the CMM (blue) predicts the relevant concept for input $x_i$

The CMM consists of (a) an encoder $g:\mathbb {R}^n \rightarrow \mathbb {R}^{512}$ that takes as input $x_i$ and outputs a vector $\widehat{{w_{i} }} \in {\mathcal {W}}$ (b) similarity block Sim(.) that retrieves closest aligning concept $w_i$ from $\mathcal {D}$ (Fig.  3). Encoder *g* is trained to predict $\widehat{{w_{i} }}$ such that the properties of the latent space remain preserved as the samples are embedded. This is done using the cost function $\mathcal {L} = \alpha \mathcal {L}_{NCE} +\beta \mathcal {L}_{MSE} + \gamma \mathcal {L}_{KL}$. $\mathcal {L}_{KL}$ minimizes the Kullback–Leibler divergence between the actual ${\textbf {p}}(w|x)$ and predicted $\hat{{\textbf {p}}}(\hat{w}|x)$ latent variable distributions (Eq. 1)

1 $$\begin{aligned} \mathcal {L}_{KL} = KL({\textbf {p}}||\hat{{\textbf {p}}}) = \sum _{x} {\textbf {p}}(w|x) log \frac{{\textbf {p}}(w|x)}{\hat{{\textbf {p}}}(\hat{w}|x)} \end{aligned}$$

However, simply ensuring that the two distributions converge is not enough to ensure that the newly embedded $\hat{w_i}$s span the latent space according to its geometrical properties. For example, input images could be mapped to the average latent $\overline{w}$ in the space regardless of the semantic content.

Furthermore, since the latent dimensions/concepts in $\mathcal {W}$ are orthogonal (disentangled) from each other, the inverse mapping from $x_i$ to $\hat{w_i}$ through *g* must be such that editing along $\hat{w_i}$ does not change aspects in the image corresponding to $[w_j]_{j!=i}$, i.e., alignment with one concept automatically encourages orthogonality with others. Therefore, we use $\mathcal {L}_{NCE}$ to enforce these constraints. Assuming concepts to be independent (orthogonal in latent space) and that each image arises from a single task-relevant concept, instances with the same concept within a batch must align strongly with the same *w* in the latent space, similarly instances from different concepts must lead to mutual orthogonality in the latent space. This leads to $\mathcal {L}_{NCE}$ which is a cross entropy loss over cosine similarities between [*w*] vectors in a batch, which simplifies to the InfoNCE objective given by:

2 $$\begin{aligned} \mathcal {L}_{NCE} = -\mathbb {E} \, log \left[ \frac{exp \frac{s(\hat{w_i},w_+)}{\tau }}{exp\frac{s(\hat{w_i},w_+ )}{\tau } + \sum _{j\in [neg]} exp \frac{s(\hat{w_i}, [w_j])}{\tau }}\right] \end{aligned}$$

where s is the cosine similarity score, $w_+, w_{neg}$ the positives and negatives for $\hat{w_i}$ and $\tau$ is the temperature parameter. Finally, $\mathcal {L}_{MSE} = {|w_i - \hat{w_i} |}^2$ is the mean squared error over the actual and predicted $w_i$. Once the encoder is trained, it can be used to retrieve the human-interpretable concept from $\mathcal {D}$. This is done by first predicting $\hat{w_i}$ for the test samples, followed by a cosine similarity check against the concepts in $\mathcal {D}$ to find the concept with maximal alignment with $\hat{w}$. Figure 1 shows our approach.

### Training details

StyelGAN2 has been trained on unlabeled WCE images. A total of 200,000 images from three sources have been used for training. Apart from datasets PS-DeVCEM [12] and OSF-Kvasir [13], images from the capsule examinations of patients with varying activity of ulcerative colitis (as well as other pathologies) with PillCam Colon2 Capsule, Medtronic, have been used [14]. Binary classifier $\mathcal {C}$ uses pretrained weights [12] for classification. $\mathcal {C}$ predicts for both real images from the datasets described above as well as generated images. Concept explanations for both types of images (real and generated) can be generated by the CMM. *g* of CMM is a Resnet-50 [15] encoder. We use StyleGAN2 with hyperparameters as in [10] and omit progressive growing in the interest of reducing computational complexity. In this experiment, the scaling factors are $\alpha = 0.6, \beta = \gamma = 0.2$. However, these values may require additional tuning depending on the application domain.

**Sampling the positives and negatives for** $\mathcal {L}_{NCE}$: As $\mathcal {L}_{NCE}$ optimizes $\hat{w}$ such that similarity with positives $w_+$ is maximized and that with the negatives $w_{neg}$ is minimized. In a batch of size B, for input $x_i$ with concept $w_i$, the positives correspond to other images with the same concept. For example, for an image with concept polyp, all other polyp images in the batch qualify as positives, while the remaining images qualify as the negatives. In this work, B=64, we sample one positive from the batch for each $w_i$ and all negative samples within the batch are used for computing $\mathcal {L}_{NCE}$.

### Concepts for capsule endoscopy

**Fig. 2:**
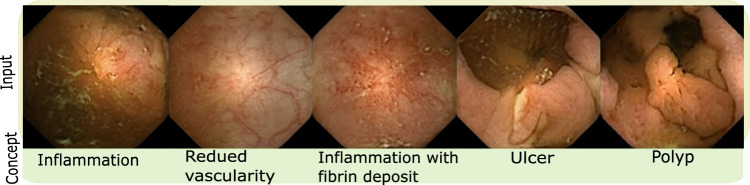
Figure shows example images for five task-related concepts discovered by proposed approach. As described previously, the concept is named (bottom row) in collaboration with a gastroeneterologist (28 years of experience) by showing him/her images corresponding to each candidate concept from the dataset

The concept exploration was performed with the help of a gastroeneterologist with 28 years of experience, whereby four task-related concepts were identified (shown in Fig. 2), which are inflammation (mild and severe), vascularity changes, ulcer and polyp. Since the images are essentially generated, a more thorough study conducting complete evaluation of data and its clinical plausibility can be found in [14]. The medical concepts in this paper were utilized only after establishing strong clinical plausibility in prior work.

**Fig. 3:**
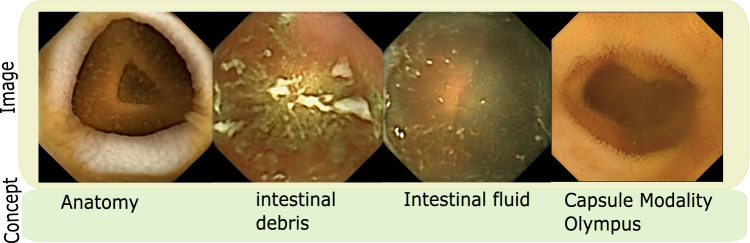
Figure shows images for some candidate concepts that were not selected for pathology classification problem discussed in the main paper

Also, not all dimensions within the latent space $\mathcal {W}$ correspond to useful or task-relevant concepts. As such, in a pathology classification, concepts relating to normalcy such as anatomical variations, image variations arising from different capsule cameras or absence or presence of gastrointestinal content are not required and hence are not included in concept dictionary $\mathcal {D}$. However, if the problem were slightly different as clean Vs occluded image classification, organ classification or capsule modality classification, these concepts would become task-relevant. Figure  3 shows images from some of these concepts.

## Results

Through the use of a concept mapper as described, the latent spaces of pretrained StyleGANs can be utilized to retrieve relevant concepts as explanations. While there are works indicating that dimensions in latent space are potent for concept curation, we formally define an approach through which concept-based explanations can be retrieved from latent space. Further, using latent dimensions as the starting point for concept curation saves both time and resources compared to starting from raw data and domain experts. The results thus far strongly motivate further exploration for further refining explanations, multiclass classifiers as well as other domains.

## Limitations

The explanations can be in terms of only those concepts that are present already within the latent space; in other words, new concepts cannot be added on the fly for explaining images that do not lie in the latent distribution. Therefore, the method is limited to images *x* for which a *w* exists not too far from the training distribution. An interesting future work in this direction is the incorporation of an uncertainty metric along with explanations that provide a measure of how much can a given explanation be trusted.
